# Basal Cell Carcinoma Masked in Rhinophyma

**DOI:** 10.1155/2013/201024

**Published:** 2013-06-11

**Authors:** Daniele De Seta, Francesca Yoshie Russo, Elio De Seta, Roberto Filipo

**Affiliations:** Sensory Organs Department, Sapienza University of Rome, Viale dell'Università 31, 00161 Rome, Italy

## Abstract

Rhinophyma, the advanced stage of rosacea, is a lesion characterized by progressive hypertrophy and hyperplasia of sebaceous glandular tissue, connective tissue, and blood vessels. Rhinophyma can lead to a significant facial disfigurement and severe emotional distress, but it is not only an aesthetic problem, since rare cases of simultaneous presence of malignant tissue are described in the literature. The case of an 84-year-old farmer affected by basal cell carcinoma (BCC) and diagnosed in the context of rhinophyma is presented. The anatomical distortion produced by the chronic inflammation and fibrous scarring makes the BCC diagnosis difficult and uncertain. The histological examination of the entire mass and its margins is fundamental. A partial biopsy can lead to a false negative result, and the histological examination must be repeated intra- or postoperatively.

## 1. Introduction

Basal cell carcinoma (BCC) represents about 75–90% of skin cancers [1, 2]. 85% of these tumors are located in the head and neck area, the majority of these affecting the nose [3]. Fair skin phenotype and sun exposure are important risk factors for this kind of tumor unlike other skin lesions. Although slow growing and unlikely to metastasize, these tumors may have an indolent course and are prone to recur if inadequately treated [1, 2]. If neglected, tumor-related destruction of anatomic features may create difficult reconstructive challenges. In aggressive forms of BCC, associated with extensive dermal invasion and destruction of collagen, the identification of tumor borders can be difficult [4]. Clinical diagnosis can be difficult as the characteristics of the carcinomatous lesions may be masked by the background of soft tissue hypertrophy and distortion seen in gross rhinophyma. Rhinophyma is a rare condition caused by a progressive hypertrophy of sebaceous glandular tissue, connective tissue, and blood vessels. It is considered to represent the final stage of severe rosacea, although it may occur in patients with few or no features of rosacea [5]. Caucasian people between 45 and 60 years old, with a male-female ratio of 5/1–30/1, are more frequently affected by rhinophyma, while it is slightly present in the black people [6]. Its etiology remains unknown, even though several causes have been proposed, including vitamin deficiencies, stress, androgenic hormone influences, and chronic infections by the *Demodex folliculorum* mite [7]. Convincing evidence for a causal relationship between malignancy and rhinophyma is currently lacking [8]; nevertheless, some cases of BCC are reported in the literature discovered at the time of a rhinophyma excision [9]. Thus, the question of whether rhinophyma may be considered as a premalignant lesion still remains unanswered.

## 2. Case Presentation

**Figure 1:**
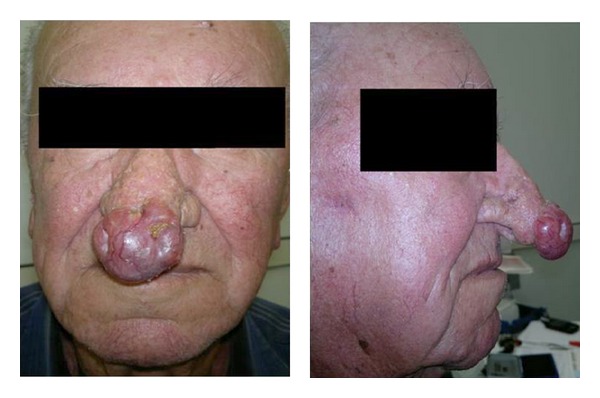
Rhinophyma at presentation.

An 84-year-old Caucasian male farmer, with blue eyes and fair complexion, was admitted in the ENT Department of Sapienza, University of Rome. The patient presented a wide rounding mass on the tip of the nose with a small ulceration and a widespread telangectasia in a typical appearance of angiomatous rhinophyma (Figure 1). The lesion was hard and not painful at the palpation; hyposmia and nasal obstruction were reported by the patient. The physical examination resulted negative for other facial lesions and cervical adenopathy. The mass increased in volume over the preceding 5 months. A peripheral biopsy of the lesion was performed in another hospital previously, laying for parakeratosis and orthokeratosis with no signs of cell dysplasia. The patient's past family history was negative for cancer and other relevant pathologies. Clinical history comprehended malaria infection contracted at the age of three and polymyalgia rheumatica treated with corticosteroid therapy during the last three years. Continuative sun exposure due to the patient's work activity and history of mild alcohol and tobacco consumption were reported.

**Figure 2:**
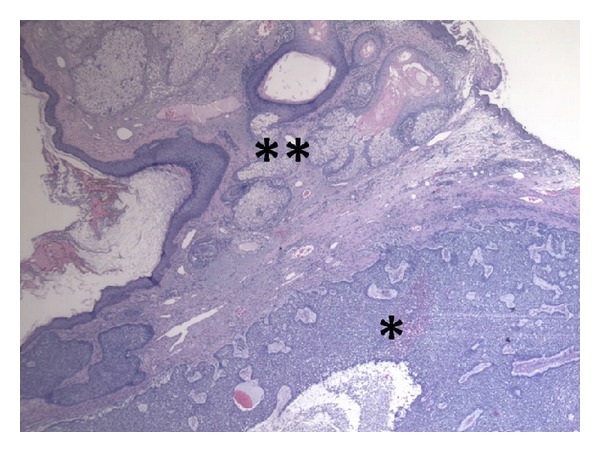
Basal cell carcinoma adjacent to rhinophyma in nasal skin—there are cystic nests of hyperchromatic and uniform basaloid cells (*) with peripheral palisading surrounded by loose stroma close to highly hypertrophic sebaceous glands (**), typical of rhinophyma.

Surgical removal was planned. The resection of the lesion was performed with a radiofrequency scalpel and sent for histological examination (Figure 2). The result of histology evidenced an invasive BCC with the typical microcystic nodular aspect, incompletely resected, and surrounded by skin with the typical appearance of rhinophyma. The size of the excised lesion was 4 × 3 × 2 cm. 

A second operation for the complete removal of the residual tumor was performed. The lesion was excised with more profound margins, and the reconstruction of the nose was performed using a full-thickness skin graft from groin area. The margins resulted negative at the new histological examination. In the followup, the patient did not present recurrence of the disease.

## 3. Discussion

The coexistence of rhinophyma and carcinoma was first described in 1904 by Wende and Bentz [10]. The authors described the case of a patient with rhinophyma of which histological examination revealed the simultaneous presence of five different tumors. In their investigation, Brubaker and Hellstom [11] hypothesized that the papillary buds emanating from the basal layer of the dilated follicles might be the primary area of the malignant degeneration. Furthermore, this area showed a range of proliferative cellular changes from hyperplasia to BCC, and they asserted that the incidence of BCC is 5% in patients with rhinophyma. Several hypotheses have been suggested to explain the association of BCC and rhinophyma. Scarring fibrous tissue, frequent in rhinophyma [4], skin trauma [5], and hypertrophic and hyperplasic cellular changes [2], have all been proposed to explain the connection between the two conditions. The present case highlights the necessity of a careful examination of the whole rhinophymatous tissue, for coexisting carcinoma. The distortion of tissues due to the hypertrophic growth of rhinophyma makes the clinical identification and the early detection of the malignancy challenging; therefore, the lesion may be underestimated for a long time. Partial biopsies can lead to a misdiagnosis; subsequently, during surgery the lesion could be removed without a safe cleaning of the margins. Clear margins during surgical removal of extended tumors may be obtained with intraoperative histological analysis. Moh's micrographic surgery could be considered, and it is asserted by some authors to be the treatment of choice for carcinoma arising within rhinophyma [12]. The present case demonstrates the importance of a rigorous monitoring of patients affected by rhinophyma and the necessity to always remove the entire rhinophymatous mass, as a BCC can be masked within the lesion. The certain diagnosis of either rhinophyma or BCC can be obtained only by the histological examination of the entire excised mass, since a partial biopsy can lead to a false negative result. A periodic followup is needed in case of BCC diagnosis.
